# Expansion of human alpha‐cell area is associated with a higher maximum body mass index before the onset of type 2 diabetes

**DOI:** 10.1111/1753-0407.13370

**Published:** 2023-02-26

**Authors:** Harutoshi Ozawa, Kenji Fukui, Yukari Fujita, Chisaki Ishibashi, Sho Yoneda, Takao Nammo, Shingo Fujita, Megu Yamaguchi Baden, Takekazu Kimura, Ayumi Tokunaga, Junji Kozawa, Hidetoshi Eguchi, Iichiro Shimomura

**Affiliations:** ^1^ Department of Metabolic Medicine, Graduate School of Medicine Osaka University Suita Japan; ^2^ Department of Community Medicine, Graduate School of Medicine Osaka University Suita Japan; ^3^ Yoneda Clinic Osaka Japan; ^4^ Department of Diabetes Care Medicine, Graduate School of Medicine Osaka University Suita Japan; ^5^ Department of Gastroenterological Surgery, Graduate School of Medicine Osaka University Suita Japan

## Abstract

**Highlights**

- We examined whether maximum body mass index (BMI) before the onset of diabetes (MBBO) affects histological findings of islet cells.
- We divided patients into two groups according to an MBBO cutoff of 25 kg/m^2^ or BMI cutoff of 21 kg/m^2^. We compared immunohistochemical parameters between the MBBO groups or BMI groups.
- The relative alpha‐cell area in the high MBBO group was significantly higher than that in the low MBBO group. There was no difference in the other parameters between the MBBO groups or BMI groups.

## 1 BACKGROUND

Type 2 diabetes is characterized by deteriorated insulin secretion capacity and abnormal glucagon secretion.^1^ In White people, beta‐cell mass correlates with body mass index (BMI),^2^ whereas alpha‐cell mass does not correlate with BMI.^3^ In Japanese patients, there was no difference in either the relative beta‐cell area or alpha‐cell area between obese and nonobese patients with or without diabetes.^4^

We reported that maximum BMI before the onset of diabetes (MBBO) independently correlated with beta‐cell function, enabling us to estimate insulin secretion capacity at onset as well as at present.^5^ However, the relationship between MBBO and relative beta‐ or alpha‐cell area remains unclear. The purpose of this study was to clarify this relationship with the use of immunohistochemical analysis using human pancreatic tissues.

## 2 METHODS

We enrolled 34 patients who had already been diagnosed with type 2 diabetes mellitus and had undergone partial pancreatic resection between 2008 and 2013 and between 2018 and 2019 in the Department of Gastroenterological Surgery, Osaka University Hospital, Suita, Japan. Along the chart described in Figure S1, we analyzed 20 Japanese patients (15 men and 5 women) in our study. The study protocol was approved by the ethics committee of Osaka University (approval number 13279‐4) and was carried out in accordance with the Declaration of Helsinki. Informed consent was obtained from all patients.

Before the operation, we conducted a medical interview including a history of the patient's body weight. We defined the patient's MBBO based on his or her history of maximum BMI and age at diabetes mellitus onset.

Beta‐cell function was evaluated before the operation using the C‐peptide index (CPI), which was calculated by using the following formula: fasting C‐peptide level (ng/mL) x 100/fasting plasma glucose level (mmol/l) x 18. We previously demonstrated significant positive correlations between the relative beta‐cell area, a factor indicating beta‐cell mass, and various parameters of insulin secretion capacity, including CPI.^6^

We obtained pancreatic tissue samples from patients who had undergone partial pancreatectomy. Normal noncancerous pancreatic samples were collected during the operation. The tissues were isolated near the resected margins after intraoperative consultation, fixed immediately in formaldehyde, and embedded in paraffin for subsequent analysis. Paraffin‐embedded tissue was cut into 5‐μm thick sections.

**FIGURE 1:**
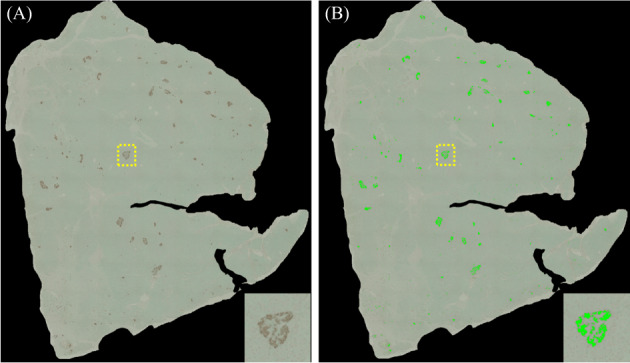
Digital calculation of beta‐cell area using WinROOF. (A) Beta‐cell areas were stained brown, using an avidin–biotin complex and a 3,3‐diaminobenzidine tetrahydrochloride substrate. Pancreatic sections were counterstained with methyl green. (B) Brown areas are marked with light green and digitally calculated.

**FIGURE 2:**
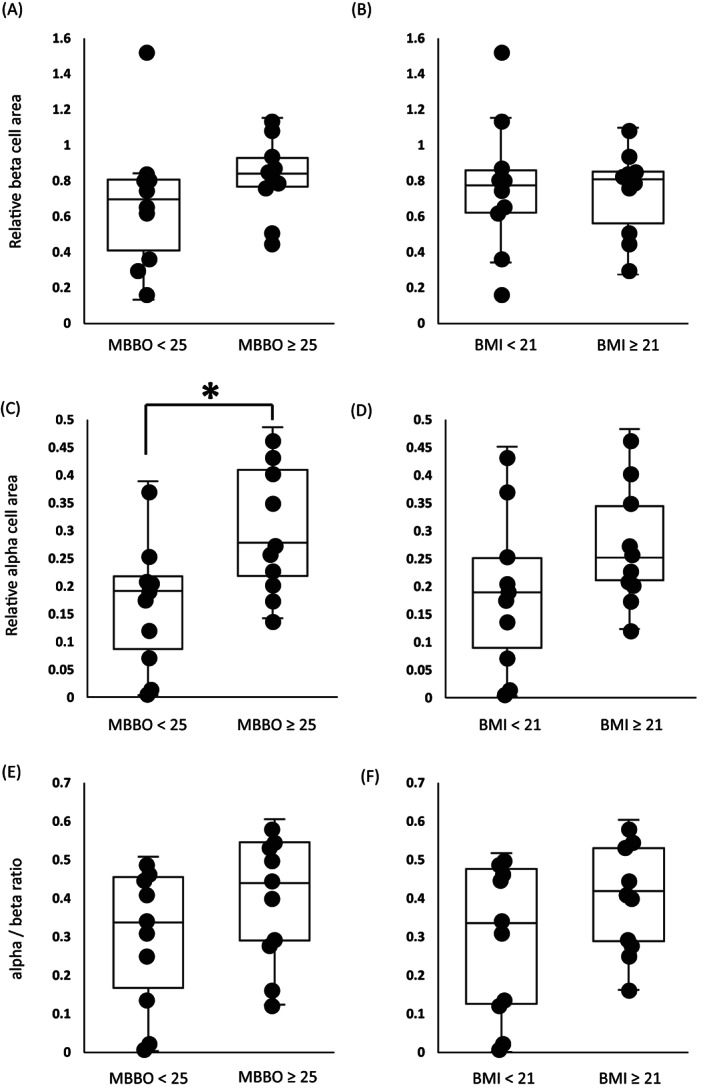
We divided patients into two groups according to an MBBO cutoff of 25 kg/m^2^ (low group: MBBO <25 kg/m^2^, *n* = 10; high group: MBBO ≥ 25 kg/m^2^, *n* = 10) (A, C, E) or BMI cutoff of 21 kg/m^2^ (low group: BMI < 21 kg/m^2^, *n* = 10; high group: BMI ≥ 21 kg/m^2^, *n* = 10) (B, D, F). We compared immunohistochemical parameters between the MBBO groups or BMI groups. The relative alpha‐cell area in the high MBBO group was significantly higher than that in the low MBBO group (C). There was no difference in the other parameters between the MBBO groups or BMI groups. *p < .05. BMI, body mass index; MBBO, maximum BMI before the onset of diabetes.

The primary and secondary antibodies and chromogenic substrates used in the present study are listed in Table S1. We stained beta cells and alpha cells using anti‐insulin and anti‐glucagon immunoglobulins (Igs) as primary antibodies and biotinylated Igs as secondary antibodies. The reactions were developed with an avidin–biotin complex and a 3,3‐diaminobenzidine tetrahydrochloride substrate, followed by methyl green counterstaining. Figure 1 shows how to calculate relative beta‐ or alpha‐cell area using stained images. As a surrogate for beta‐ and alpha‐cell mass, the beta‐ and alpha‐cell areas were determined by the proportion of insulin‐positive or glucagon‐positive cell area relative to the whole pancreatic section (%; Figure 2A–D), which were quantified digitally with the WinROOF software program (Mitani Corporation, Fukui, Japan). We also calculated the alpha‐ to beta‐cell area (alpha/beta) ratio (Figure 2E,F), which is higher in patients with type 2 diabetes than in nondiabetic subjects.^4^, ^7^

Immunohistochemical analyses were carried out on one section per patient. The median area of the sections used to determine the relative beta‐ and alpha‐cell area was 26.4 and 24.8 mm^2^.

Data collected in our study were nonnormally distributed, and these data are presented as the medians and interquartile ranges (IQRs). The data were compared using the Wilcoxon test. *p* values <.05 denoted the presence of a statistically significant difference. All statistical analyses were carried out with the JMP Pro 14 software program (Statistical Analysis System Inc., Cary, NC, USA).

## 3 RESULTS

The clinical characteristics of the subjects are shown in Table 1. The median MBBO was 24.9 kg/m^2^ (IQR, 22.7 to 28.5), and the median glycated hemoglobin (HbA1c) was 6.7% (IQR, 6.4 to 7.1). The median relative beta‐cell area was 0.79% (0.53, 0.86), the median relative alpha cell area was 0.21% (0.15, 0.33), and the alpha/beta ratio was 0.37 (0.18, 0.48). Primary diseases were mainly pancreatic carcinoma (*n* = 7), intraductal papillary mucinous carcinoma (*n* = 1), and cystic lesions of the pancreas (*n* = 7), including intraductal papillary mucinous neoplasm, mucinous cystic neoplasm, and simple cyst. Other diseases included cholangiocarcinoma (*n* = 1), tumor of the ampulla of Vater (*n* = 3), and pancreatic metastasis from renal cell carcinoma (*n* = 1). Between admission and operation, all of the patients received insulin therapy using a rapid‐acting insulin analog for glycemic control.

**TABLE 1:** Clinical characteristics of the patients.

	ALL	MBBO < 25	MBBO ≥ 25	*p* value
Age (years)	71 (65, 81)	73 (69, 80)	69 (65, 82)	.57
Sex (M/F)	15/5	8/2	7/3	.61
Duration (years)	13 (1, 27)	13 (1, 24)	13 (1, 32)	.88
BMI on admission (kg/m^2^)	20.8 (19.2, 23.2)	19.8 (18.1, 21.5)	22.1 (20.6, 23.8)	.076
MBBO (kg/m^2^)	24.9 (22.7, 28.5)	22.9 (20.3, 23.9)	27.7 (25.6, 30.9)	.0002
HbA1c (%)	6.7 (6.4, 7.1)	6.6 (6.3, 6.9)	6.9 (6.5, 7.4)	.27
HbA1c (mmol/mol)	49.7 (46.7, 53.8)	48.1 (45.6, 52.2)	51.4 (47.3, 57.6)	.27
Glucose (mmol/l)	6.6 (5.6, 7.4)	6.5 (5.1, 7.4)	6.7 (5.8, 9.9)	.41
CPI (*n* = 16)	1.01 (0.52, 1.38)	1.05 (0.56, 1.56)	0.62 (0.48, 1.1)	.53
Operative procedure (PD/DP)	15/5	5/5	10/0	.033
Medication before admission				
Sulfonylurea (%)	5 (25%)	2 (20%)	3 (30%)	.61
Glinide (%)	1 (5%)	1 (10%)	0 (0%)	.30
Alpha‐GI (%)	4 (20%)	2 (20%)	2 (20%)	1.00
DPP4i (%)	6 (30%)	4 (40%)	2 (20%)	.33
Insulin (%)	7 (35%)	3 (30%)	4 (40%)	.64
No medication (%)	6 (30%)	4 (40%)	2 (20%)	.33

*Note*: Data are reported as the median (interquartile range) or *n* (%), unless otherwise indicated. Comparisons between the two groups divided by MBBO were performed by a Wilcoxon test or a *χ* ^2^ test for data presented as the median (interquartile range) or *n* (%), respectively. *p* values <0.05 were considered statistically significant.

Abbreviation: Alpha‐GI, alpha‐glucosidase inhibitor; BMI, body mass index; CPI, C‐peptide index; DP, distal pancreatectomy, DPP4i, dipeptidyl peptidase‐4 inhibitor; HbA1c, glycated hemoglobin; MBBO, maximum BMI before onset; PD; pancreatoduodenectomy.

We divided patients into two groups according to the MBBO cutoff of 25 kg/m^2^ (low group: MBBO < 25 kg/m^2^, *n* = 10; high group: MBBO ≥ 25 kg/m^2^, *n* = 10, Table 1). There was a significant difference in operative procedures between the MBBO groups. However, there was no difference in any other clinical characteristics between these two groups.

The relative alpha‐cell area in the high MBBO group was significantly higher than that in the low MBBO group (0.26 [IQR 0.19 to 0.41] vs. 0.18 [IQR 0.057 to 0.22], *p* = .031, Figure 2C), while the relative beta‐cell area was not different between these two groups (0.83 [IQR 0.69 to 0.97] vs 0.70 [IQR 0.34 to 0.81], *p* = .15, Figure 2A). The alpha/beta ratio was not different between MBBO groups (0.42 [IQR 0.25 to 0.53] vs. 0.32 [IQR 0.11 to 0.45], *p* = .24, Figure 2E). Then, we divided patients into two groups according to a BMI cutoff of 21 kg/m^2^ (low group: BMI < 21 kg/m^2^, *n* = 10; high group: BMI ≥ 21 kg/m^2^, *n* = 10). There was no difference in these histological parameters between the two groups (Figure 2B,D,F).

## 4 CONCLUSIONS

This is the first report that showed that a high MBBO group was associated with a high relative alpha‐cell area, while MBBO was not associated with a high relative beta‐cell area. Our alpha‐cell area results were different from those reported by previous studies,^3^, ^4^ which could not observe an association between alpha‐cell area and BMI. In those studies, BMI was defined independent of the onset of diabetes and could be affected by hypoglycemic agents. We identified the difference in relative alpha‐cell area among patients with type 2 diabetes focusing on past maximum BMI before the onset of diabetes.

One of the possible mechanisms of the increase in alpha‐cell mass in patients with higher MBBO is the proliferation of alpha cells, which is induced by amino acids.^8^ Increased branched‐chain amino acid derived from meal digestion stimulates glucagon secretion from alpha cells^9^ and flow into hepatocytes.^10^ Secreted glucagon binds to glucagon receptors in the liver and increases hepatic amino acid catabolism.^11^ However, some conditions, such as fatty liver disease, cause glucagon resistance, which is explained by decreased expression of hepatic glucagon receptor,^12^ leading to increased blood amino acid levels.^13^ High blood amino acid levels result in alpha‐cell expansion.^14^ Patients with higher MBBO might have had fatty liver as well as the intake of a large amount of amino acids.

We hypothesize that the length of the history of obesity may be associated with the degree of alpha‐cell hypertrophy. Patients with higher MBBO had higher alpha‐cell mass, suggesting that higher MBBO patients might have a longer period of obesity. It would be necessary to investigate the detailed change in body weight of the patients to confirm our hypothesis.

In our study relative beta‐cell area was not different between MBBO groups or BMI groups, while our previous study showed a significant correlation between MBBO and insulin secretary capacity.^5^ The enhanced computed tomography before the operation showed that the patient with the highest relative beta‐cell area in the low MBBO group had pancreatic head carcinoma and had very thin pancreatic parenchyma as well as dilated pancreatic duct (Figure S2), though there was no obvious history of pancreatitis. As a result, relative beta cell area of this patient would be relatively large because of atrophied pancreatic exocrine region. In fact, the patient had low insulin‐secreting capacity (CPI = 0.45) despite the large beta‐cell area. This may be because patients with high MBBO had similar beta cell area as those with low MBBO.

This study had some limitations. First, alpha‐cell proliferation could not be evaluated when the BMI of the patients reached MBBO. Second, the sample size was relatively small. Third, this is a retrospective study, and the preoperative routine examinations did not include measurements of serum insulin or glucagon levels. Thus, we could not obtain these data at the time of operation as well as the time of MBBO. We could not confirm the relationships between pancreatic endocrine functions and histological findings of these islet cells. Finally, patients who underwent pancreatectomy were included in the present study, and it is possible that the pancreatic histological findings of islet cells were affected by the underlying diseases.

In conclusion, the high MBBO group had a high relative alpha‐cell area, while there was no difference in the relative beta‐cell area between the MBBO groups. We identified a difference in relative alpha‐cell area among Japanese patients with type 2 diabetes according to MBBO.

## AUTHOR CONTRIBUTIONS

Harutoshi Ozawa, Kenji Fukui, and Junji Kozawa designed the whole project and wrote the manuscript. Harutoshi Ozawa contributed to the acquisition and analysis of the data. Hidetoshi Eguchi examined the patients and obtained pancreatic tissue samples. Chisaki Ishibashi, Shingo Fujita, and Yukari Fujita assisted with the data analysis and reviewed and edited the manuscript. Sho Yoneda, Takekazu Kimura, and Junji Kozawa assisted with the study design and reviewed and edited the manuscript. Takao Nammo and Ayumi Tokunaga reviewed and edited the manuscript. Megu Yamaguchi Baden provided statistical advice regarding the study design and assisted with the data analysis. Iichiro Shimomura assisted with the study design and analysis and reviewed and edited the manuscript. All authors revised the manuscript critically for important intellectual content and approved the final version of the manuscript. Junji Kozawa is the guarantor of this work and, as such, had full access to all the data in the study and takes responsibility for the integrity of the data and accuracy of data analysis.

## CONFLICT OF INTEREST STATEMENT

The authors declare no conflicts of interest.

## Supporting information


**FIGURE S1.** Flow chart for the recruitment of the patients.

**FIGURE S2.** Abdominal enhanced computed tomography of the patient with the highest relative beta‐cell area in the low MBBO.

**TABLE S1.** Primary antibodies and secondary antibodies used.

## Data Availability

The data sets generated and analyzed during the current study are available from the corresponding author on reasonable request.
