# Supplementary figures and images for: Expansion of human alpha‐cell area is associated with a higher maximum body mass index before the onset of type 2 diabetes
Source: J Diabetes. 2023 Feb 26;15(3):277–82. doi: 10.1111/1753-0407.13370 (PMC10036255; doi:10.1111/1753-0407.13370)

# 1    **Supplementary Figure 1**

## 2    **Flowchart for the recruitment of the patients**

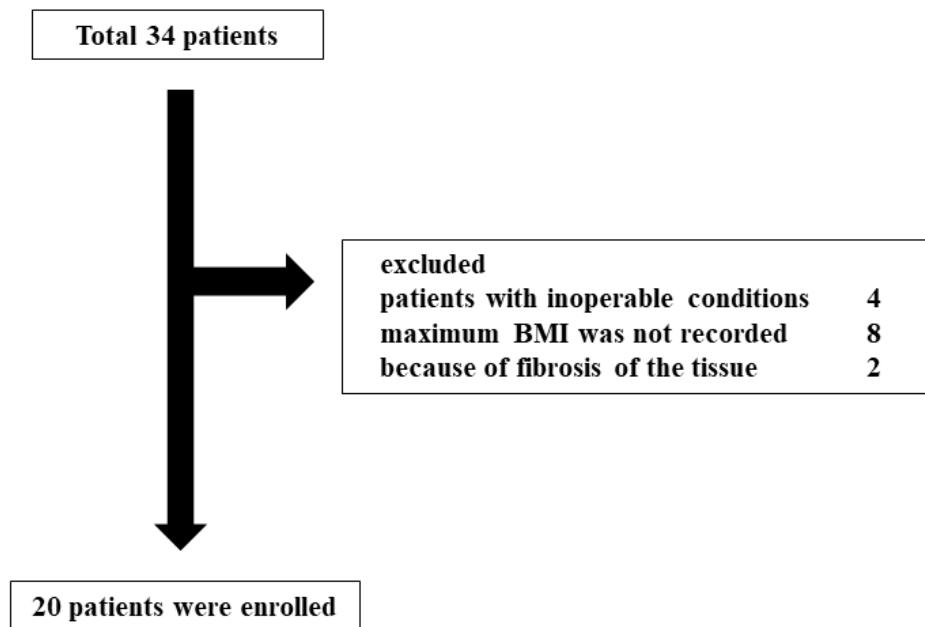

Supplement: Supplementary file 1 — FIGURE S1. Flow chart for the recruitment of the patients. [file JDB-15-277-s003.pdf]
