# Supplementary material for: Expansion of human alpha‐cell area is associated with a higher maximum body mass index before the onset of type 2 diabetes
Source: J Diabetes. 2023 Feb 26;15(3):277–82. doi: 10.1111/1753-0407.13370 (PMC10036255; doi:10.1111/1753-0407.13370)

Supplementary Figure 2

Abdominal enhanced computed tomography of the patient with the highest relative beta-cell area in the low MBBO

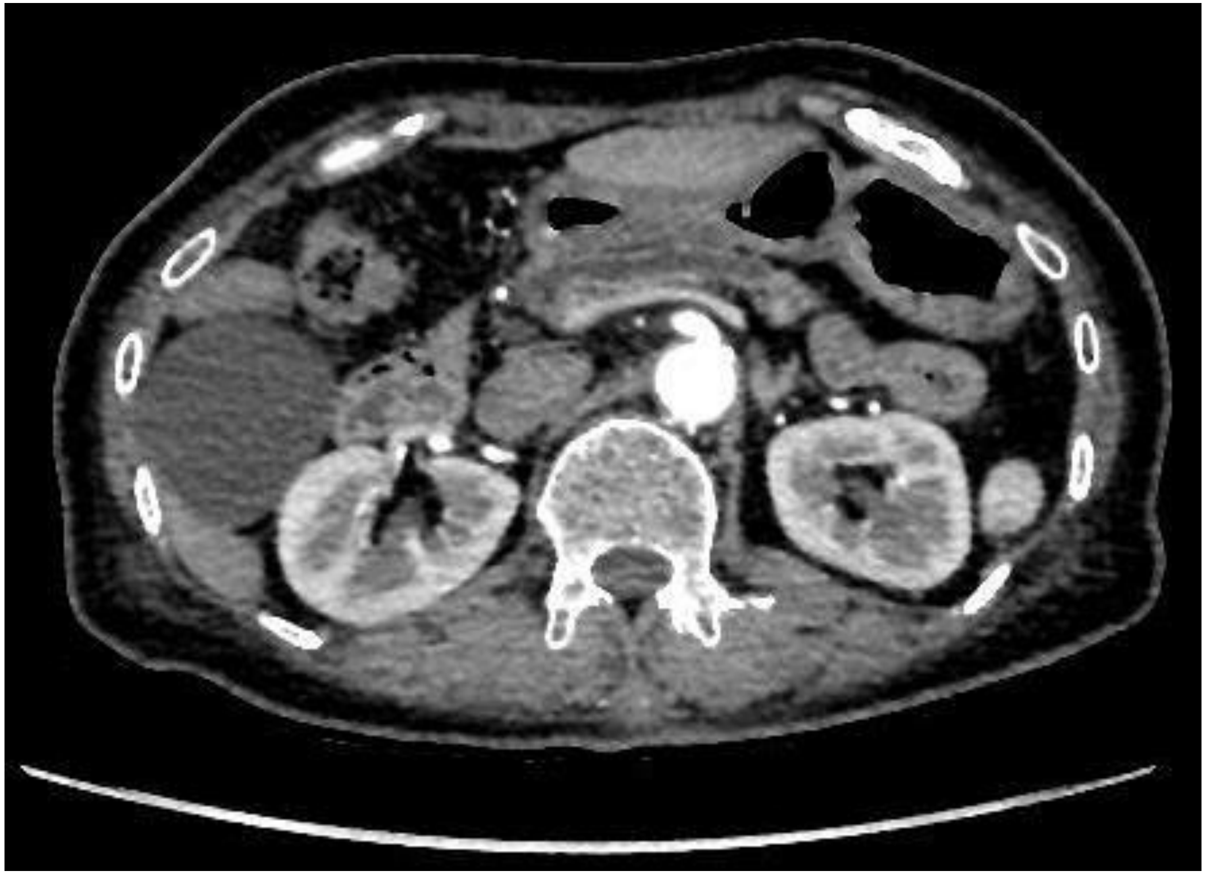

Supplement: Supplementary file 2 — FIGURE S2. Abdominal enhanced computed tomography of the patient with the highest relative beta‐cell area in the low MBBO. [file JDB-15-277-s002.pdf]
