# Supplementary material for: Expansion of human alpha‐cell area is associated with a higher maximum body mass index before the onset of type 2 diabetes
Source: J Diabetes. 2023 Feb 26;15(3):277–82. doi: 10.1111/1753-0407.13370 (PMC10036255; doi:10.1111/1753-0407.13370)
Supplement: Supplementary file 3 — TABLE S1. Primary antibodies and secondary antibodies used. [file JDB-15-277-s001.pdf]

Supplementary Table 1

Primary antibodies and secondary antibodies used

Primary antibodies

Antigen	Species	Source	RRID	Dilution
Insulin	Guinea pig	Dako Japan, Kyoto, Japan	AB_10013624	1:1000
Glucagon	Mouse	Sigma, Missouri, USA	AB_259852	1:500

Secondary antibodies

Antigen	Species	Source	RRID	Dilution
Guinea pig	Goat (biotinylated)	Vector Laboratories, California, USA	AB_233613	1:200
Mouse	Horse (biotinylated)	Vector Laboratories, California, USA	AB_2313581	1:200
